# Knockout of integrin β1 in induced pluripotent stem cells accelerates skin-wound healing by promoting cell migration in extracellular matrix

**DOI:** 10.1186/s13287-022-03085-7

**Published:** 2022-07-30

**Authors:** Yansong Ren, Jinbo Liu, Huijun Xu, Shun Wang, Shirui Li, Meng Xiang, Sifeng Chen

**Affiliations:** Department of Physiology and Pathophysiology, School of Basic Medical Sciences, Fudan University, Shanghai, 200032 People’s Republic of China

**Keywords:** Induced pluripotent stem cells, Wound-healing, Integrin β1, Cell adhesion, Cell migration

## Abstract

**Background:**

Induced pluripotent stem cells (iPSCs) have the potential to promote wound healing; however, their adhesion to the extracellular matrix (ECM) might decrease iPSC migration, thereby limiting their therapeutic potential. Integrin β1 (Itgb1) is the major integrin subunit that mediates iPSC-ECM adhesion, suggesting that knocking out *Itgb1* might be an effective method for enhancing the therapeutic efficacy of iPSCs.

**Methods:**

We knocked out *Itgb1* in mouse iPSCs and evaluated its effects on the therapeutic potential of topically applied iPSCs, as well as their underlying in vivo and in vitro mechanisms.

**Results:**

The *Itgb1*-knockout (*Itgb1-*KO) did not change iPSC pluripotency, function, or survival in the absence of embedding in an ECM gel but did accelerate wound healing, angiogenesis, blood perfusion, and survival in skin-wound lesions. However, embedding in an ECM gel inhibited the in vivo effects of wild-type iPSCs but not those of *Itgb1-*knockout iPSCs. Additionally, in vitro results showed that *Itgb1-*knockout decreased iPSC-ECM adhesion while increasing ECM-crossing migration. Moreover, ECM coating on the culture surface did not change cell survival, regardless of *Itgb1* status; however, the in vivo and in vitro functions of both *Itgb1*-knockout and wild-type iPSCs were not affected by the presence of agarose gel, which does not contain integrin-binding sites. Knockout of Integrin α4 (Itga4) did not change the above-mentioned cellular and therapeutic functions of iPSCs.

**Conclusions:**

*Itgb1*-knockout increased iPSCs migration and the wound-healing-promoting effect of topically applied iPSCs. These findings suggest the inhibition of Itgb1 expression is a possible strategy for increasing the efficacy of iPSC therapies.

**Supplementary Information:**

The online version contains supplementary material available at 10.1186/s13287-022-03085-7.

## Background

Wound-healing delays and failures seriously affect patient recovery and appearance [1]. Stem cells can differentiate into cutaneous tissue cells, promote angiogenesis, and secrete beneficial substances [2, 3], thereby helping to promote wound healing. However, due to the adhesion of stem cells to the extracellular matrix (ECM), their limited migration in wounds subsequently limits their therapeutic efficacy [4, 5].

Although stem cells are migratory cells, their migration in the interstitial space is mediated by the type and number of cell-adhesion molecules expressed on their surface [6, 7]. To achieve therapeutic efficacy, stem cells should home to the site where they are needed. The first step is extravasation, adhering to and migrating across the vascular wall. For mouse iPSCs, this step is facilitated by the interaction of integrin α4β1 on iPSCs with VCAM-1 on activated endothelial cells (ECs); the β1 subunit is the dominant β subunit in mouse iPSCs. In the second step, iPSCs migrate through capillary basement membranes and interstitial spaces [8]. Cell–ECM interactions are usually mediated by members of the integrin family of adhesion molecules and transmembrane receptors [9]. As cell-adhesion molecules that were initially found on leukocytes [10], integrins bind a variety of extracellular ligands, including cell receptors, ECM proteins, soluble proteins in multiple body fluids, and microbial proteins and carbohydrates [11]. The ECM ligands of the integrin β1 (Itgb1) subunit include proteins such as collagens, fibronectin, laminin, and vitronectin [12]. We previously found that Itgb1 is the most abundant β subunit expressed on the surface of induced pluripotent stem cells (iPSCs) [8]. Therefore, we hypothesized that Itgb1 mediates iPSC binding to the ECM to regulate cell adhesion and migration.

Integrins are composed of noncovalently linked α subunits and β subunits that combine to produce at least 24 different heterodimers [9]. VLA-4 (α4β1) is unique among integrins, because it is the only integrin heterodimer containing the β1 submit that binds to the ligand VCAM-1 to mediate leukocyte rolling, transendothelial cell migration, and binding to the ECM. To determine whether the amount of α unit affects the function of β1-mediated iPSC–ECM interaction, iPSCs with *Itgα4*-knockout (*Itga4*-KO) were also tested.

As angiogenesis and recovery of blood flow precede wound healing [13, 14], promoting angiogenesis is one of the mechanisms by which stem cells facilitate tissue repair [15]. In the present study, we established *Itgb1*-knockout iPSCs to investigate the role of Itgb1 in iPSC-mediated wound healing and its underlying mechanisms.

## Methods

### Generation and culture of murine iPSCs

As described previously [16], iPSCs were generated from fibroblasts isolated from embryonic day 13.5 embryos from C57BL/6 mice using an antibiotic-resistant retrovirus, containing the Tet-On transactivator, which expresses Krüppel-like factor 4 (*Klf4*), octamer-binding transcription factor 3/4 (*Oct3/4*), sex-determining region Y-box 2 (*Sox2*), and *c-Myc*. Embryonic fibroblasts (5 × 10^5^) were incubated in Dulbecco's modified Eagle medium (DMEM, high-glucose; #SH30243.01; HyClone Laboratories, Logan, UT, USA) containing 10% fetal bovine serum (FBS; #Gibco-1932597; Thermo Fisher Scientific, Waltham, MA, USA) in Petri dishes overnight; then, the medium was replaced with an equal volume of retrovirus medium supplemented with 10 µg/mL polybrene (Sigma-Aldrich, St. Louis, MO). After 24 h of transduction, the medium was replaced with fresh DMEM containing 10% FBS for an additional 48 h of culture before the addition of iPSC medium. The iPSCs were identified based on morphology, embryoid body (EB) formation, alkaline phosphatase (AP) staining, expression of pluripotency genes [(*Sox2*, *Oct4*, and *stage-specific embryonic antigen-1* (*Ssea1*)], and teratoma formation.

The iPSCs were seeded on 0.2% gelatin-coated Petri dishes and cultured in iPSC medium in a humidified culture incubator at 37 °C and 5% CO_2_. The iPSC medium was DMEM-supplemented with 10% FBS, 1% nonessential amino acid (NEAA) solution (#11140050; Thermo Fisher Scientific), 1% penicillin–streptomycin mixture (#P1400; Solarbio Life Science, Beijing, China), 1% amphotericin B (#A8250; Solarbio Life Science), 50 μM β-mercaptoethanol (#97622; Sigma-Aldrich, St Louis, MO, USA), 0.5 μM mirdametinib (PD0325901; #S1036; Selleck Chemicals, Shanghai, China), 0.5 μM CHIR-99021 (CT99021) HCl (#S2924; Selleck Chemicals), and 1 × 10^7^ U/mL recombinant mouse leukemia inhibitory factor (LIF; #ESG1107; Sigma-Aldrich). Upon reaching 80% confluence, iPSCs were detached from dishes using 0.25% trypsin–EDTA solution (#25200056; Thermo Fisher Scientific) and resuspended in iPSC medium for subsequent experiments.

### Construction of *Itgb1*- and *Itga4*-knockout iPSCs

An *Itgb1* CRISPR was designed using an online tool (https://zlab.bio/guide-design-resources) as described previously [8]. Oligonucleotides corresponding to *Itgb1* were obtained from Integrated DNA Technologies (Coralville, IA, USA) and included the sense 5'-ATGGTGTGTAGCTAGGCTAATG-3' and anti-sense 5'- TGGACTGACACTCTGCTTTG -3'. Single-guide (sg) RNA oligos were annealed and ligated to the Cas9 Lenti CRISPR version 2.0 vector that had been digested with *Bbs*I to generate the individual guide plasmid, which coexpressed Cas9 and the sgRNA. The CRISPR vectors were prepared and packaged according to the manufacturer’s instructions. iPSCs were infected with the CRISPR retrovirus vector and cultured in iPSC medium containing 10 μg/mL puromycin. Single-cell clones were generated from these transfected iPSCs and were expanded after 10–14 days of transduction. Mismatch loss of *Itgb1* was confirmed by DNA sequencing and pairwise dot matrix comparison. Successful *Itgb1*-knockout iPSCs were confirmed by PCR and western blot.

*Itgα4*-knockout iPSCs were prepared and identified using the same methods as described for *Itgβ1*-knockout iPSCs. The only differences were the sequences of single-guide (sg) RNA oligos: 5'-GCTGCTGCACTTCATCTCTT-3' and anti-sequence 5'-GGAGGGCGGAAAGTTTGATT-3'.

### EB-formation assay

To evaluate spontaneous differentiation potential, iPSC colonies were dissociated and transferred to a 0.2% gelatin-coated Petri dish and then cultured for spontaneous differentiation in EB culture medium for 7 days. The EB culture medium contained DMEM/nutrient mixture F-12 (#10565018; Thermo Fisher Scientific) with 10% KnockOut serum replacement (#10828010; Thermo Fisher Scientific), 10% 1 mM GlutaMAX supplement (#35050061; Thermo Fisher Scientific), 1% NEAA solution (#11140050; Thermo Fisher Scientific), 10% penicillin–streptomycin mixture (#10378016; Thermo Fisher Scientific), and 50 μM β-mercaptoethanol (#97622; Sigma-Aldrich). Theoretically, the formation of EBs that include differentiated cells from three embryonic germ layers from iPSCs can be observed.

### Alkaline phosphatase (AP) assay

Staining of iPSC colonies was performed using the AP detection kit (#SCR004; Sigma-Aldrich) according to the manufacturer’s instructions; AP activity can be identified by red staining.

### Immunofluorescence staining for pluripotency markers

iPSC colonies were fixed in 4% paraformaldehyde for 15 min, washed with phosphate-buffered saline (PBS), and subsequently blocked with 5% donkey serum (#017-000-001; Jackson ImmunoResearch, West Grove, PA, USA) at 25 °C for 1 h. For cytoplasmic protein detection, iPSCs were permeabilized with 0.3% Triton X-100 for 10 min and incubated with the following primary antibodies diluted in 5% donkey serum: rabbit polyclonal anti-Oct4 (1:100; #ab19857; Abcam, Cambridge, UK), rabbit polyclonal anti-Sox2 (1:100; #ab97959; Abcam), and mouse monoclonal anti-Ssea1 (1:100; #ab16285, Abcam), respectively, at 4 °C overnight. After three washes with PBS, iPSCs were incubated with Alexa Fluor 594-conjugated donkey anti-rabbit IgG (H + L) secondary antibody (1:2000; #711-585-152; Jackson ImmunoResearch) or Alexa Fluor 488-conjugated donkey anti-mouse IgG secondary antibody (1:2000; #715-545-150; Jackson ImmunoResearch) in the dark at 25 °C for 1 h. After three washes with PBS, 1 μg/mL 4',6-diamidino-2-phenylindole (#C1005; Beyotime, Beijing, China) was used to stain nuclei. After washing with PBS, iPSC colonies were sealed and observed under a fluorescence microscope (BX51; Olympus, Tokyo, Japan).

### Total RNA isolation and reverse transcription (RT)-PCR

RNA from skin wounds was isolated using TRIzol reagent (#15596026; Life Technologies, Carlsbad, CA, USA) according to the manufacturer’s instructions. RNA concentrations and quality were determined by absorbance using a NanoDrop spectrophotometer (Implen GmbH, Munich, Germany) at 260/280 nm. RT-PCR was performed using an input of 1 µg total RNA using the First Strand cDNA synthesis kit (#FSK-100; TOYOBO Bio-Technology, Osaka, Japan). Quantitative PCR was performed using cDNA and real-time PCR master mix (#QPK-201; TOYOBO Bio-Technology) in an ABI 7300 real-time PCR system (Applied Biosystems, Foster City, CA, USA) with the profile: 95 °C for 5 min, followed by 40 cycles of amplification at 95 °C for 20 s, 60 °C for 20 s, and 72 °C for 20 s. RT-PCR was performed according to the manufacturer’s instructions, with *Actb* used as the reference gene, and each sample was run in triplicate. Data were normalized to *Actb* expression, and relative expression was calculated using the 2^−ΔΔCt^ method. PCR primers were designed and confirmed by Integrated DNA Technologies (https://sg.idtdna.com/pages):

**Table:** 

Primer	Sense	Antisense
*Actb*	GTGACGTTGACATCCGTAAAGA	GCCGGACTCATCGTACTCC
*Cd31*	CAAGGCCAAACAGAAACCCG	GGAGCCTTCCGTTCTTAGGG
*Cd34*	TCATGTCCGGCCTTCTCCTA	TTGGCAGCTCCTCTAGACCT
*FoxA2*	AACCTCCCTACTCGTACATCTC	AGGTCCATGATCCACTGATAGA
*Tbx6*	TACCCGACCGTGTCTACAT	TGTGCTGTTGGTGAGCTT
*Nes*	AGAAGCAGGGTCTACAGAGT	GTATGTAGCCACTTCCAGACTAAG

### Animals

Male C57BL/6 mice (4–8-weeks old, 20–25 g) were purchased from Shanghai SLAC Laboratory Animal Co., Ltd. (Shanghai, China) and maintained at the Medical Laboratory Animal Center of Shanghai Medical College, Fudan University (Shanghai, China).

### Teratoma-formation assay

Aliquots of 5 × 10^6^ iPSCs in 50 µL of Matrigel (#356234; BD Biosciences, San Jose, CA, USA) per site were injected subcutaneously into C57BL/6 mice. Four weeks after injection, the mice were euthanized and tumors confirmed using hematoxylin/eosin staining and RT-PCR to determine whether teratomas contained tissues from all three germ layers.

### Protein isolation and Western blotting

Total protein from tissue lysates of iPSCs or skin tissues was isolated using Radio-immunoprecipitation assay buffer (#89901; Thermo Fisher Scientific) containing 1% protease-inhibitor cocktail and 1% phenylmethylsulfonyl fluoride according to the manufacturer’s instructions. Proteins were separated by 10% sodium dodecyl sulfate–polyacrylamide gel electrophoresis and transferred onto a polyvinylidene difluoride (PVDF) membrane (Immobilon-P; Millipore, Bedford, MA, USA). The membrane was blocked for 1 h in 10% skim milk and then incubated with primary antibodies against integrin β1 (1:100; #sc-374429; Santa Cruz Biotechnology, Dallas, TX, USA) and β-actin (1:10,000; #AB0035; Abways, Shanghai, China) overnight at 4 °C. The following day, the membrane was washed three times for 10 min each with PBS containing 0.1% Tween-20 and then incubated with Peroxidase-AffiniPure donkey anti-rabbit IgG (H + L) (1:2000; #711-035-152; Jackson ImmunoResearch) at 37 °C for 1 h, followed by three washes for 10 min each with PBS. The immunoreactive bands were visualized using a chemiluminescence substrate detection system (Tanon, Beijing, China).

### Establishment of the full-thickness excisional cutaneous-wound model and iPSC treatments

Before creation of a full-thickness excision-wound model, mice were anesthetized by intraperitoneal injection of sodium pentobarbital (30 mg/kg body weight) after induction of sedation by an intramuscular injection of ketamine hydrochloride (10 mg/kg body weight). The dorsal skin was shaved and removed with a depilatory cream (Veet; Reckitt Benckiser, Massy, France), the central axis of the dorsal skin was lifted, and two symmetrical full-thickness cutaneous wounds were produced using 4-mm-diameter biopsy punchers (BD Pharmingen, San Diego, CA, USA). The mice received topical applications of PBS, wild-type (WT)-iPSCs, *Itgb1*^−/−^-iPSCs, agarose-embedded WT-iPSCs, agarose-embedded *Itgb1*^−/−^*-*iPSCs, Matrigel-embedded WT-iPSCs, Matrigel-embedded *Itgb1*^−/−^-iPSCs, *Itgα4*^−/−^-iPSCs, agarose-embedded *Itgα4*^−/−^*-*iPSCs, or Matrigel-embedded *Itgα4*^−/−^-iPSCs immediately after creation of the wound (*n* = 10 mice/topical application). We used 1 × 10^4^ iPSCs in 20 μL PBS, 20 μL 1% low-melting-point agarose, or 20 μL 1% Matrigel for topical application to the skin wound, with PBS (20 μL) without cells used as the control. The mice were euthanized on days 6 and 12 after wound creation, and wound tissue from dorsal skin was excised. The wound tissue was divided equally: half was stored at − 80 ℃ for the use of RNA and DNA solution, and the other half was fixed with 4% paraformaldehyde for frozen sectioning.

### Survival of fluorescent iPSCs

To track iPSC fates in vivo, additional experiments were performed with the lesions having topical applications of PBS, wild-type (WT)-iPSCs, *Itgb1*^−/−^-iPSCs, *Itgα4*^−/−^-iPSCs, agarose-embedded WT-iPSCs, agarose-embedded *Itgb1*^−/−^*-*iPSCs, agarose-embedded *Itgα4*^−/−^*-*iPSCs Matrigel-embedded WT-iPSCs, Matrigel-embedded *Itgb1*^−/−^-iPSCs, and Matrigel-embedded *Itgα4*^−/−^-iPSCs (*n* = 3). All cells were labeled with PKH26 as described below. Lesions were monitored and scored as described for the experiment with nonlabeled cells. Furthermore, the same experiments were performed using iPSCs expressing GFP instead of PKH26-fluorescence labeled iPSCs. *n* = 3.

### Cell labeling with PKH26 Red

iPSCs were labeled using the PKH26 Red fluorescent cell linker kit (#PKH26GL; Sigma-Aldrich) according to the manufacturer’s instructions prior to their use. Cells (1 × 10^7^) were dissociated using 0.25% trypsin–EDTA, washed with DMEM without FBS, and resuspended in 500 μL diluent C from the cell linker kit to prepare a 2 × cell suspension. PKH26 cell linker reagent (2 μL) was diluted in another volume of 500 μL diluent C as working solution, mixed thoroughly, then incubated at room temperature for 5 min. Staining was stopped by adding an equal volume of FBS and then incubation for 1 min to allow binding of excess reagent. After washing with PBS, cells were resuspended for subsequent processing.

### Lentiviral GFP transfection of iPSCs

The GFP plasmids (pCMV-N-EGFP, with CMV promoter) were amplified and cloned into lentivirus constructs to generate a GFP plasmid vector. Then, the lentiviral expression constructs and the packaging plasmid mixture, including pGag/Pol, pRev, and pVSVG, were cotransfected into human 293 T cells. The GFP plasmid vectors were obtained from the supernatant at 48 and 72 h, respectively. Mouse iPSCs were transfected with these vectors and then cultured for 2 weeks in iPSC medium supplemented with 0.5 mg/ml puromycin to select for GFP-expressing iPSCs.

### Wound-closure evaluation

The day of wound creation was designated as day 0. Wounds were monitored for 14 days, with images captured on days 0, 2, 4, 6, 8, 10, and 12. Wound areas were measured using ImageJ software (v.1.51 k; National Institutes of Health, Bethesda, MD, USA), and the percentage of wound closure is calculated using the equation:

$${\text{wound}}\;{\text{healing }}\;{\text{percentage}}\;{\text{ (\% ) = }}\frac{{{\text{wound}}\;{\text{area}}\;{\text{on }}\;{\text{day }}\;{\text{0 - wound}}\;{\text{area}}\;{\text{on }}\;{\text{the }}\;{\text{indicated }}\;{\text{day}}}}{{{\text{wound }}\;{\text{area}}\;{\text{ on }}\;{\text{day}}\;{ 0}}} \times 100\% .$$

### Blood perfusion measurement

Perfusion in skin tissues was measured using serial scanning with a laser Doppler imaging system (MoorLDI2-HIR, high-resolution; Moor Instruments, Axminster, UK) on days 0, 2, 4, 6, 8, 10, and 12 after wound creation. The scanning parameters were: Scan Size: Normal; Scan Area (unit): X0 = 108, Y0 = 104, dX = 41, dY = 41; Scan Resolution (pixel): X = 41,Y = 41; Scan Speed: 4 ms/pixel. Laser Doppler signals were quantified using regions of interest (same size between measurements) and are presented as relative perfusion units (PUs; a relative unit defined against a controlled motility standard).

### Production of frozen sections

Tissues were fixed with 4% paraformaldehyde for 24 h and then dehydrated through 15%, 20% and 30% continuous sucrose gradients. The dehydrated tissues were embedded in Tissue-Tek O.C.T. Compound (#4583, SAKURA, Finetek, USA) to ease handling and then continuously sectioned at 5 μm at − 20 °C using a Tissue-Tek cryostat (SAKURA, Tokyo, Japan).

### Fluorescence microscopy

Frozen sections prepared from the skin lesions receiving iPSCs labeled with PKH26 or overexpressing GFP were observed and photographed with an inverted fluorescence microscope (Leica, Wetzlar, Germany) with DAPI (4′,6-diamidino-2-phenylindole) counterstaining of nuclei.

### Immunohistochemistry (IHC)

Frozen sections were subjected to antigen retrieval using 10-mM sodium citrate buffer (pH 6.0) at 95 °C for 40 min, cooled naturally for 30 min, and washed three times with tap water for 3 min. Sections were then incubated with 0.3% hydrogen peroxide for 15 min to block endogenous peroxidase activity, followed by washing with PBS. Sections were incubated with blocking solution (5% normal donkey serum in PBS) for 1 h at 25 °C, followed by overnight incubation at 4 °C with rabbit monoclonal anti-CD31 (1:100; #ab222783; Abcam, UK) diluted in blocking solution and washing with PBS. Sections were incubated with peroxidase-AffiniPure donkey anti-rabbit IgG (H + L) (1:5000; #711-035-152; Jackson ImmunoResearch) at room temperature for 1 h, followed by washing with PBS. Peroxidase activity was visualized using diaminobenzidine (#CW2069; CoWin Bioscience Co., Beijing, China). Sections were counterstained with hematoxylin and mounted with neutral balsam mounting medium. For the negative control, the primary antibody was replaced with blocking solution. Images were captured using a microscope (BX51; Olympus). Vessels in skin tissues were assessed by IHC staining for CD31 in five areas of vascular density per section of skin tissue (× 400 magnification). Signal densities were measured using ImageJ software (v.1.51 k; National Institutes of Health), with the average signal density per section of skin tissue (× 400 magnification) considered as the vessel density. The pathologists were blinded to all clinical data and group divisions.

### Quantification of iPSCs in wound tissues

*Tet-on* is a noncoding gene that can be carried into iPSCs by retroviral vectors and used for iPSC induction but not expressed in recipient cells, making it useful as a marker for counting iPSC quantification. We isolated genomic DNA from wound tissues, using DNAzol reagent (#10503027; Thermo Fisher Scientific) according to the manufacturer’s instructions. Each sample was subjected to PCR in triplicate. PCR products were separated and detected using 2.5% agarose gel electrophoresis. Intensities of PCR bands were measured using ImageJ software (v.1.51 k; National Institutes of Health), with 18S rDNA PCR products were used as internal controls to quantify *Tet-on* expression. The following *Tet-on*- and 18S rDNA-specific PCR primers were designed and confirmed by Integrated DNA Technologies (https://sg.idtdna.com/pages): *Tet-on* sense, 5'-AGCACAACTACGCCGCACCC-3' and antisense, 5'- ATGCACCAGAGTTTCGAAGC-3'; and 18S sense, 5'-GAGAAACGGCTACCACATCC-3' and antisense, 5'-CACCAGACTTGCCCTCCA-3'.

### Cell adhesion assay

Matrigel was slowly thawed at 4 °C and added to a 96-well plate at a concentration of 50 µL/cm^2^ using pre-cooled pipette tips. We then added 1% low-melting-point agarose (#75510019; Thermo Fisher Scientific) to another 96-well plate at a concentration of 50 µL/cm^2^ to coat the plate as a nonprotein matrix control for Matrigel. The 96-well plates were incubated at 37 °C for 30 min to allow polymerization, after which a suspension of PKH26-labeled iPSCs (1 × 10^3^) was seeded onto uncoated, Matrigel, and agarose plates, followed by incubation for another 2 h. Nonadherent cells were washed away with PBS; then, adherent iPSCs were counted using fluorescence microscopy. The experiment was performed using five replicates and repeated three times.

### Cell-migration assay

Matrigel (50 µL/cm^2^) was added to the upper side of the basement membrane of a Transwell chamber (#353097; Falcon cell culture inserts for 12-well plates with 8-μm membrane pores; Corning, Corning, NY, USA) using pre-cooled pipette tips; in addition, 1% low-melting-point agarose (50 μL/cm^2^) was added in the same way as a nonprotein matrix control for Matrigel. The chambers were then incubated at 37 °C for 30 min to allow polymerization, and the basement membrane was hydrated before use. A PKH26-labeled iPSC suspension (1 × 10^4^ cells) was used to seed the upper side of the basement membrane, followed by incubation for 36 h. Nonmigrating cells on the upper side of the basement membrane in the Transwell chambers were washed away with PBS; then, migrated iPSCs were counted using fluorescence microscopy. The experiment was performed using three replicates and repeated three times.

### Cell proliferation assay

Matrigel (50 µL/cm^2^) was added to a 96-well-plate using pre-cooled pipette tips, and 1% low-melting-point agarose (50 µL/cm^2^) was added to another 96-well plate as a nonprotein matrix control for the Matrigel, followed by incubation at 37 °C for 30 min to allow polymerization. Plates were seeded with iPSCs (5 × 10^3^ cells/well) in culture medium. Proliferation was measured using Cell Counting Kit-8 (CCK-8) reagent (#CKD4; Dojindo Laboratories, Kumamoto, Japan) according to the manufacturer’s instructions. Absorbance at 450 nm was measured using a microplate spectrophotometer (BioTek, Winooski, VT, USA) at 0, 24, 48, 72, and 96 h after seeding. The experiment was performed using six replicates and repeated three times.

### Apoptosis assay

Cells were scored using a BD FACSCalibur flow cytometer (BD Biosciences, San Jose, CA, USA) and an annexin V/FITC apoptosis detection kit (#AD10-10; Dojindo Laboratories), with 10,000 events acquired and analyzed using Cell Quest Software (BD Biosciences). iPSCs were cultured on plates that were uncoated or precoated with Matrigel or agarose for 48 h, followed by detachment of the iPSCs using trypsin to prepare a single-cell suspension and transfer of 2 × 10^5^ cells to a flow tube for washing with 2 mL PBS. Cells were resuspended in 200 µL of 1 × Annexin-V binding buffer to prepare a suspension at 1 × 10^6^ cells/mL. We then added 5 μL of Annexin-V/FITC solution and 10 μL propidium iodide-staining solution to the suspension, which was incubated in the dark for 15 min. Cells were analyzed within 1 h by flow cytometry. The experiment was repeated three times. Data were analyzed using FlowJo software (v.10.0; FlowJo LLC, Ashland, OR, USA). Annexin V-FITC^−^/PI^+^(Q1), annexin V-FITC^+^/PI^+^(Q2), annexin V-FITC^+^/PI^−^(Q3) and annexin V-FITC^−^/PI^−^(Q4) were necrotic, late apoptotic, early apoptotic and living cells, respectively. Their percent rates out of total cells (Q1 + Q2 + Q3 + Q4) were calculated, respectively.

### Statistical analysis

Data are presented as mean ± standard deviation, with an unpaired *t-*test used for comparisons between two correspondent groups. *p* < 0.05 was considered significant.

## Results

### *Itgb1-*knockout iPSCs

**Fig. 1:**
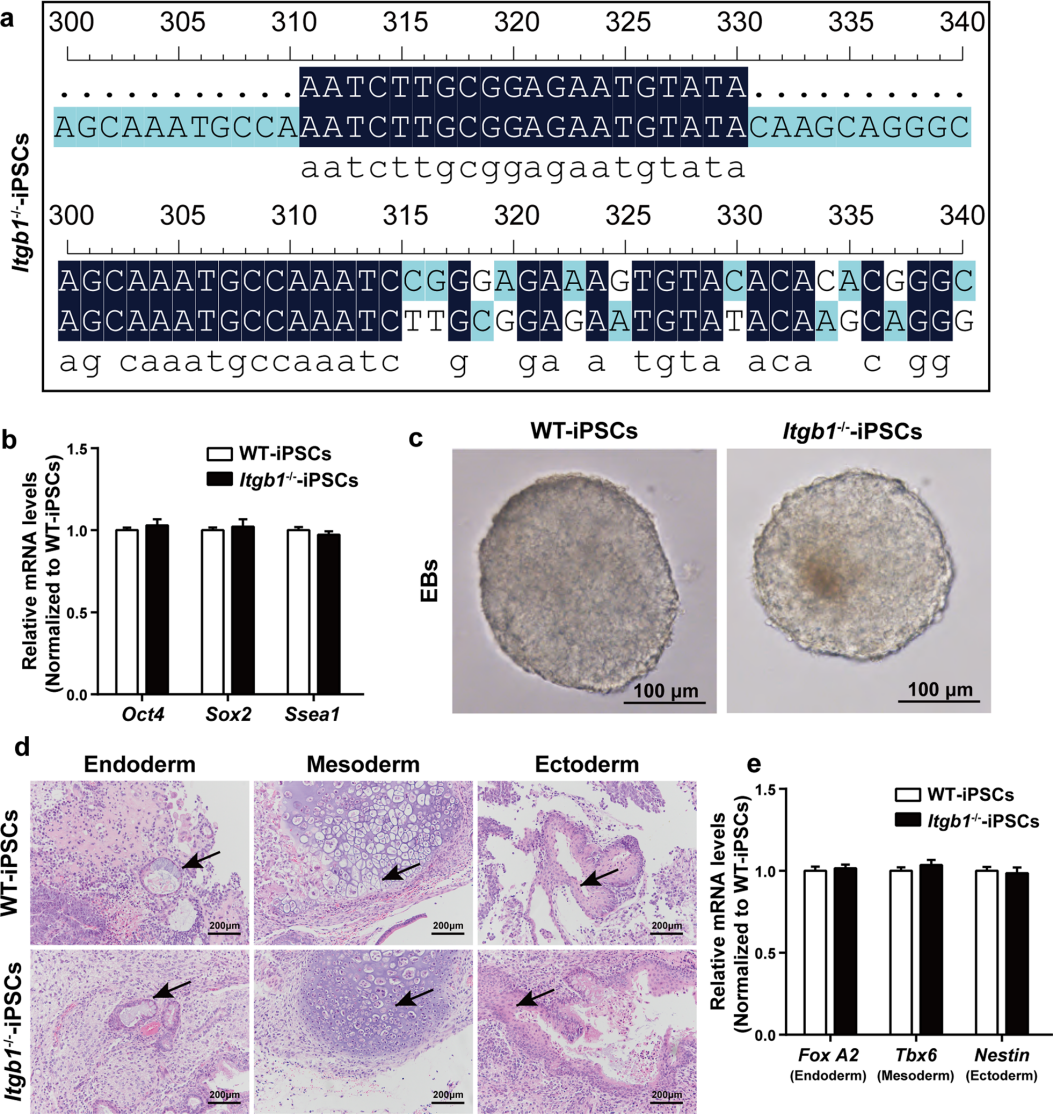
Induction and characterization of *Itgb1-*knockout iPSCs. **a** Pairwise dot matrix sequence comparison showing mismatch loss. **b**–**e** *Itgb1*^−/−^-iPSCs expressed pluripotency mRNA and were capable of forming EBs and teratomas

We generated murine *Itgb1-*knockout iPSCs (*Itgb1*^−/−^-iPSCs) using CRISPR-Cas9 as described previously [8, 16]. The knockout was confirmed by DNA sequencing. Prior to this study, we had performed additional DNA sequencing [8] and pairwise dot-matrix comparisons (Fig. 1a) to confirm the mismatch loss in *Itgb1*^−/−^-iPSCs. We observed no significant differences between *Itgb1*^−/−^-iPSCs and WT-iPSCs in general biological characteristics, including embryonic formation, AP activity, and cell proliferation, apoptosis, adhesion, migration, and expression of pluripotency markers, as well as teratoma formation [8].

### Absence of ITGß1 accelerates wound healing by iPSCs

**Fig. 2:**
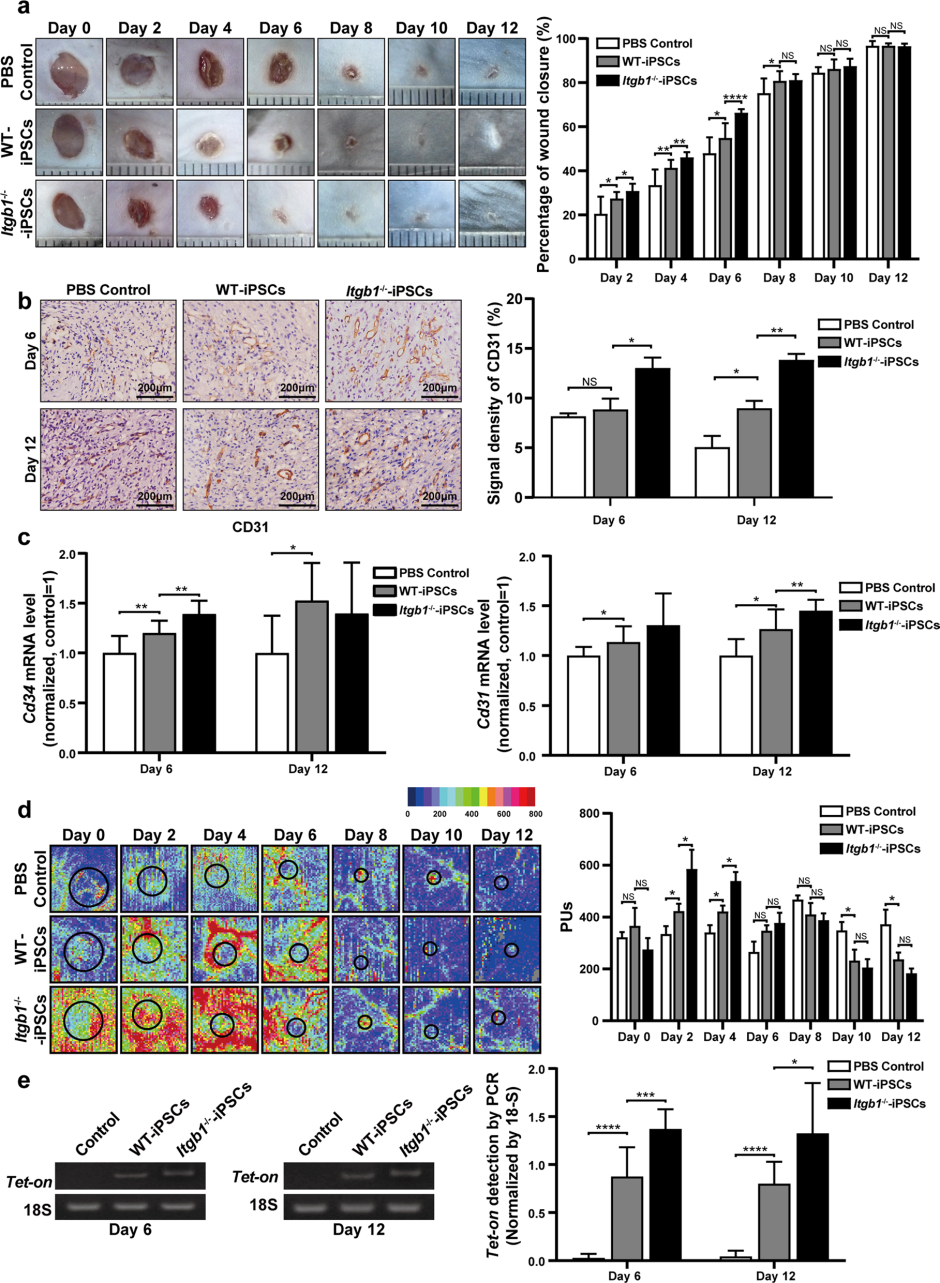
Knockout of *Itgb1* accelerates wound healing and iPSC survival in skin wound tissues. **a** Representative macroscopic images of cutaneous wounds treated with iPSCs or an equal volume of PBS on days 0, 2, 4, 6, 8, 10, and 12 post-treatment (left). Quantification of wound healing expressed as the percentage of healing relative to initial wound size at day 0 (right) (*n* = 10). **b** Representative photomicrographs of CD31 IHC staining of wound tissues on days 6 and 12 post-treatment (left). The CD31 signal density was determined using ImageJ software (right) (*n* = 5). **c** Quantification of *Cd34* and *Cd31* mRNA levels in wound tissues (*n* = 10). **d** Representative blood perfusion images of cutaneous wounds treated with iPSCs or an equal volume of PBS on days 0, 2, 4, 6, 8, 10, and 12 post-treatment (left). Quantification of blood perfusion in wound tissues (right) (*n* = 10). **e** Representative agarose gel electrophoresis images of *Tet-On* and 18S DNA (left). Quantification of *Tet-on* expression normalized to 18S expression in wound tissues (right) (*n* = 10). *p < 0.05, **p < 0.01, ***p < 0.001, ****p < 0.0001

We created a full-thickness excisional cutaneous-wound model on the dorsal skin of mice, followed by topical transplantation of iPSCs or an equal volume of PBS as a control. Macroscopic evaluation was performed at two-day intervals after wounding. We found that the wound healing with WT-iPSC treatment was significantly more rapid than that with the PBS control on days 2 (27.44 ± 2.81% vs. 20.58 ± 7.32%), 4 (41.38 ± 3.37% vs. 33.56 ± 6.72%), 6 (54.96 ± 6.29% vs. 48.08 ± 6.73%), and 8 (80.86 ± 4.16% vs. 75.25 ± 6.25%) post-treatment. More importantly, wound healing with *Itgb1*^−/−^-iPSC treatment was significantly more rapid than that in the WT-iPSC group on days 2 (30.83 ± 3.11% vs. 27.44 ± 2.81%), 4 (46.15% ± 2.15% vs. 41.38 ± 3.37%), and 6 (66.39 ± 1.49% vs. 54.96 ± 6.2%) post-treatment (Fig. 2a).

### Absence of ITGß1 promotes wound angiogenesis and blood perfusion

On days 6 and 12 post-treatment, wound tissues were collected. IHC detection of CD31 showed that localization primarily in the membrane and cytoplasm of positive cells, with CD31 signal density after WT-iPSC treatment significantly higher than that in PBS controls on day 12 (8.98 ± 1.49% vs. 5.08 ± 2.23%) post-treatment. More importantly, the CD31 signal after *Itgb1*^−/−^-iPSC treatment was significantly higher than that with WT-iPSC treatment on days 6 (13.02 ± 2.10% vs. 8.87 ± 2.15%) and 12 (13.84 ± 1.19% vs. 8.98 ± 1.49%) (Fig. 2b).

We then used RT-PCR on wound tissues to measure relative expression levels of *Cd34* and *Cd31*. *Cd34* expression after WT-iPSC treatment was significantly higher than that in PBS controls on days 6 (1.20 ± 1.17 vs. 1.00 ± 0.16) and 12 (1.52 ± 0.36 vs. 1.00 ± 0.35) post-treatment. Relative expression of *Cd34* after *Itgb1*^−/−^-iPSC treatment was significantly higher than that with WT-iPSC treatment on day 6 (1.39 ± 0.13 vs. 1.20 ± 1.17) post-treatment (Fig. 2c, left panel). Moreover, the expression of *Cd31* after WT-iPSC treatment was significantly higher than that in PBS controls on days 6 (1.14 ± 0.15 vs. 1.00 ± 0.08) and 12 (1.27 ± 0.19 vs. 1.00 ± 0.15) post-treatment. Relative expression of *Cd31* after *Itgb1*^−/−^-iPSC treatment was significantly higher than that with WT-iPSC treatment on day 12 (1.45 ± 0.11 vs. 1.27 ± 0.19) (Fig. 2c, right panel).

We also performed serial laser Doppler imaging of the dorsal skin at 2 days post-wounding. Numbers of blood PUs with WT-iPSC treatment were significantly higher than those in PBS controls on days 2 (423.19 ± 85.55 PUs vs. 334.69 ± 90.22 PUs) and 4 (421.40 ± 68.11 PUs vs. 340.68 ± 84.22 PUs) post-treatment. More importantly, blood PUs after *Itgb1*^−/−^-iPSC treatment were significantly higher than those after WT-iPSC treatment on days 2 (585.61 ± 196.00 PUs vs. 423.19 ± 85.55 PUs) and 4 (539.11 ± 103.53 PUs vs. 421.40 ± 68.11 PUs) post-treatment (Fig. 2d).

### Absence of ITGß1 increases iPSC survival in wounds

To assess in vivo iPSC survival, we quantified the expression of the *Tet-on* gene carried into iPSCs by the retrovirus used for iPSC induction in wound tissues on days 6 and 12 post-treatment. *Tet-on* expression after *Itgb1*^−/−^-iPSC treatment was significantly higher than that after WT-iPSC treatment on days 6 (1.37 ± 0.19 *vs* 0.88 ± 0.29) and 12 (1.32 ± 0.50 *vs* 0.80 ± 0.22) post-treatment (Fig. 2e).

### Absence of ITGß1 does not affect proliferation, apoptosis, adhesion, or migration in vitro in the absence of Matrigel

**Fig. 3:**
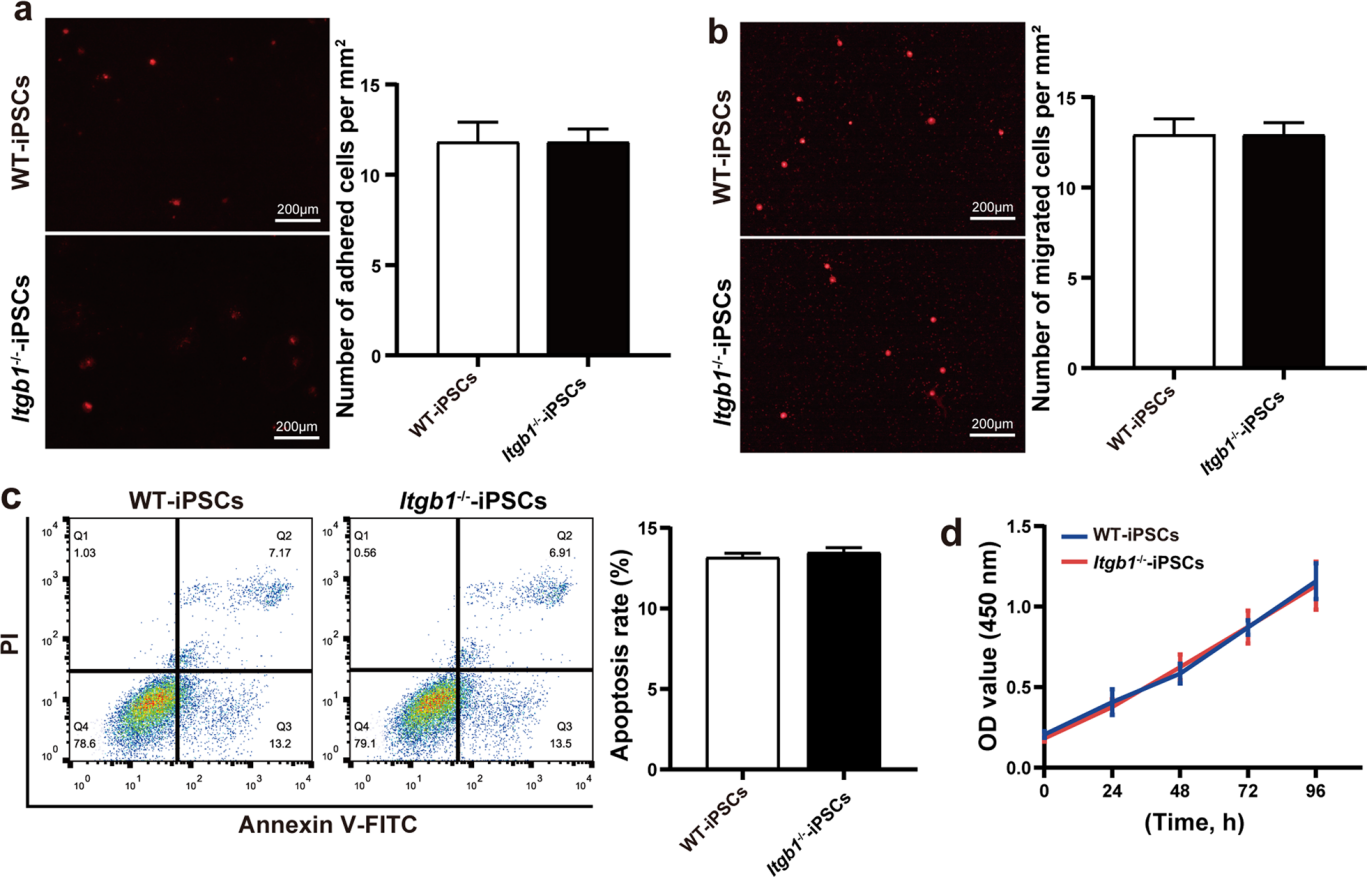
Effects of *Itgb1*-knockout on iPSCs function and survival in the absence of Matrigel. **a** Representative fluorescence imaging (left) and quantification (right) of PKH26-labeled iPSCs adhered to plates without Matrigel (*n* = 5). **b** Representative fluorescence imaging (left) and quantification (right) of PKH26-labeled iPSCs migration across the chamber membrane in the absence of Matrigel (*n* = 3). **c** iPSC apoptosis in the absence of Matrigel measured by Annexin V/FITC staining (*n* = 3). **d** iPSC proliferation in the absence of Matrigel measured by CCK-8 assay (*n* = 6). *p* < 0.05, **p < 0.01, ***p < 0.001. OD, optical density; PI, propidium iodide

We then assessed adhesion and -migration capabilities of WT and *Itgb1-*knockout iPSCs. We found no significant differences in either capability (Fig. 3a, b). To assess in vitro iPSC survival, we measured apoptosis and proliferation of WT- and ITGβ1^−/−^-iPSCs in the absence of Matrigel. We found no significant difference in either activity between *Itgb1*^−/−^- and WT-iPSCs (Fig. 3c, d).

### iPSC adhesion to Matrigel and migration are ITGß1-dependent

**Fig. 4:**
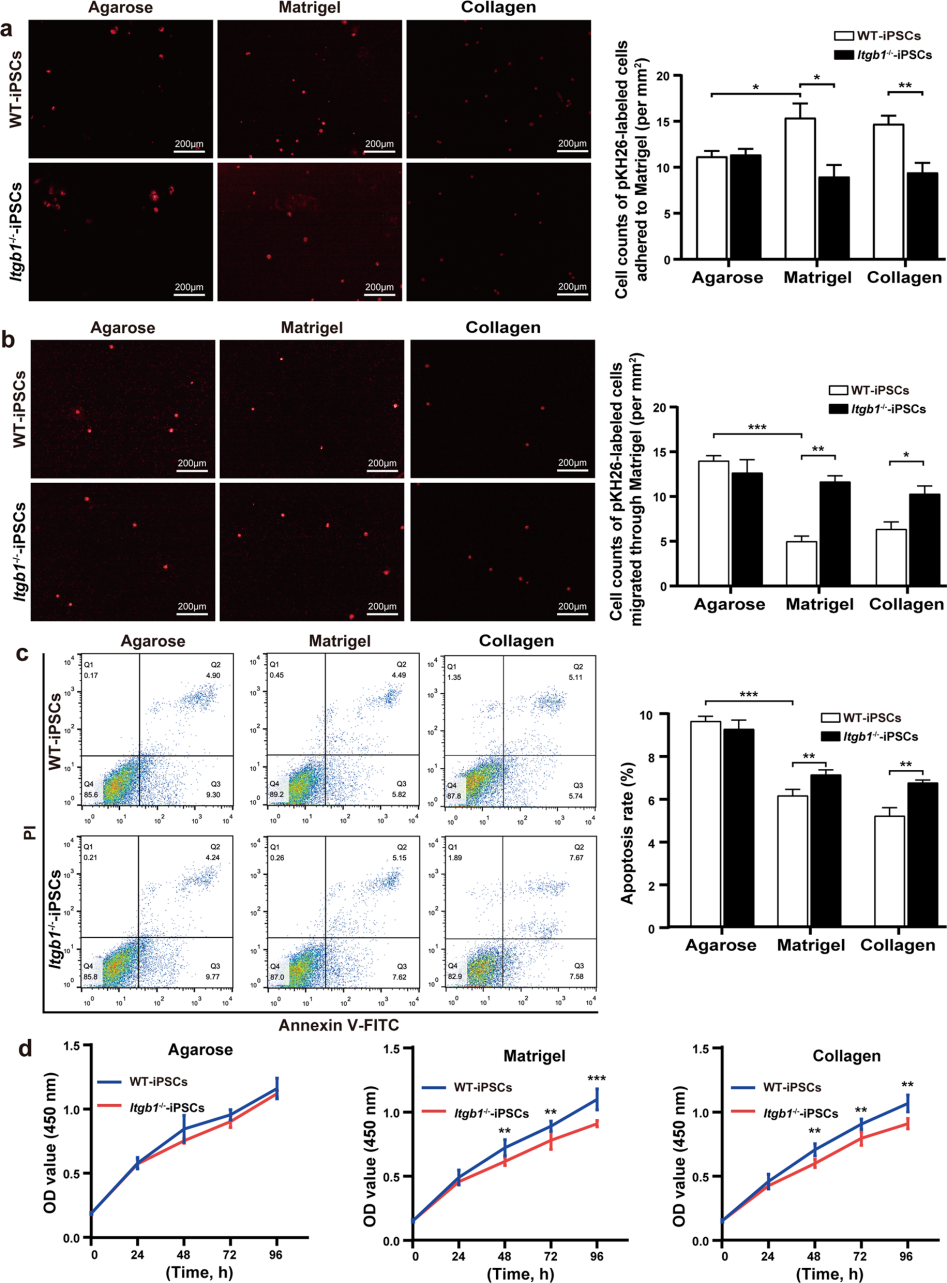
Effects of *Itgb1*-knockout on the function and survival of iPSCs cultured on Matrigel. **a** Representative fluorescence imaging (left) and quantification (right) of PKH26-labeled iPSCs adhered to plates with agarose, collagen and Matrigel (*n* = 5). **b** Representative fluorescence imaging (left) and quantification (right) of PKH26-labeled iPSC migration across the chamber membrane in the presence of agarose, collagen and Matrigel (*n* = 3). **c** Levels of iPSCs early apoptosis in the presence of agarose, collagen and Matrigel were determined by Annexin V/FITC staining (*n* = 3). **d** iPSC proliferation in the presence of agarose, collagen and Matrigel determined by CCK-8 assay (*n* = 6). *p* < 0.05, **p < 0.01, ***p < 0.001. OD, optical density; PI, propidium iodide

Collagen is the primary ligand for integrin β1 in the ECM. We tested the hypothesis that adhesion and migration in the ECM are the key factors responsible for the treatment effect. The numbers of *Itgb1*^−/−^-iPSCs that adhered to Matrigel in 2 h were significantly lower than the numbers of WT-iPSCs (9.00 ± 2.53 cells/mm^2^ vs. 15.40 ± 3.07 cells/mm^2^). The numbers of *Itgb1*^−/−^-iPSCs that adhered to collagen in 2 h were significantly lower than the numbers of WT-iPSCs (9.30 ± 2.02 cells/mm^2^ vs. 14.57 ± 2.39 cells/mm^2^). By contrast, using agarose as a nonprotein control matrix for Matrigel revealed no significant difference between genotypes. However, the numbers of WT-iPSCs that adhered to Matrigel in 2 h were significantly higher than the numbers that adhered to agarose (15.40 ± 3.07 cells/mm^2^ vs. 11.20 ± 1.16 cells/mm^2^), whereas there was no significant difference for *Itgb1*^−/−^-iPSCs between Matrigel and agarose (Fig. 4a).

The numbers of *Itgb1*^−/−^-iPSCs that migrated across Matrigel in 36 h were significantly higher than the numbers of WT-iPSCs (11.67 ± 0.94 cells/mm^2^ vs. 5.00 ± 0.81 cells/mm^2^). The numbers of *Itgb1*^−/−^-iPSCs that migrated across collagen in 36 h were significantly higher than the numbers of WT-iPSCs (10.64 ± 1.25 cells/mm^2^ vs. 6.46 ± 1.33 cells/mm^2^). We observed no significant difference between *Itgb1*^−/−^- and WT-iPSCs in their migration across agarose in 36 h. The numbers of WT-iPSCs that migrated across Matrigel in 36 h were significantly lower than the numbers that migrated across agarose (5.00 ± 0.81 cells/mm^2^ vs. 14.0 ± 0.82 cells/mm^2^), whereas there was no significant difference in migration of *Itgb1*^−/−^-iPSCs between Matrigel and agarose (Fig. 4b).

### Absence of ITGß1 reduces in vitro iPSC survival

We measured proliferation and apoptosis of WT- and *Itgb1*^−/−^-iPSCs cultured on Matrigel. We observed decreased early apoptosis in WT-iPSCs relative to *Itgb1*^−/−^-iPSCs (5.83 ± 0.91% vs. 7.94 ± 0.86%). When cultured on collagen, the result was similar, with early apoptotic rates of 4.84 ± 1.22% vs. 7.54 ± 0.48% for WT- and *Itgb1*^−/−^-iPSCs, respectively. No difference was observed when iPSCs were cultured on agarose (Fig. 4c). The change pattern of total apoptosis was similar to early apoptosis because the difference of late apoptosis among the treatments was insignificant.

The proliferation of *Itgb1*^−/−^-iPSCs was significantly decreased relative to that of WT-iPSCs at 48 h (0.63 ± 0.03 vs. 0.73 ± 0.06), 72 h (0.79 ± 0.06 vs. 0.91 ± 0.04), and 96 h (0.93 ± 0.02 vs. 1.12 ± 0.08) when cultured on Matrigel. When cultured on collagen, the proliferation data of WT-iPSCs and *Itgb1*^−/−^-iPSCs were at 48 h (0.60 ± 0.03 vs. 0.65 ± 0.04), 72 h (0.82 ± 0.03 vs. 0.95 ± 0.04), and 96 h (0.94 ± 0.04 vs. 1.10 ± 0.06), whereas no significant difference was observed for agarose (Fig. 4d).

### Absence of ITGß1 rescues Matrigel-mediated inhibition of accelerated wound healing

**Fig. 5:**
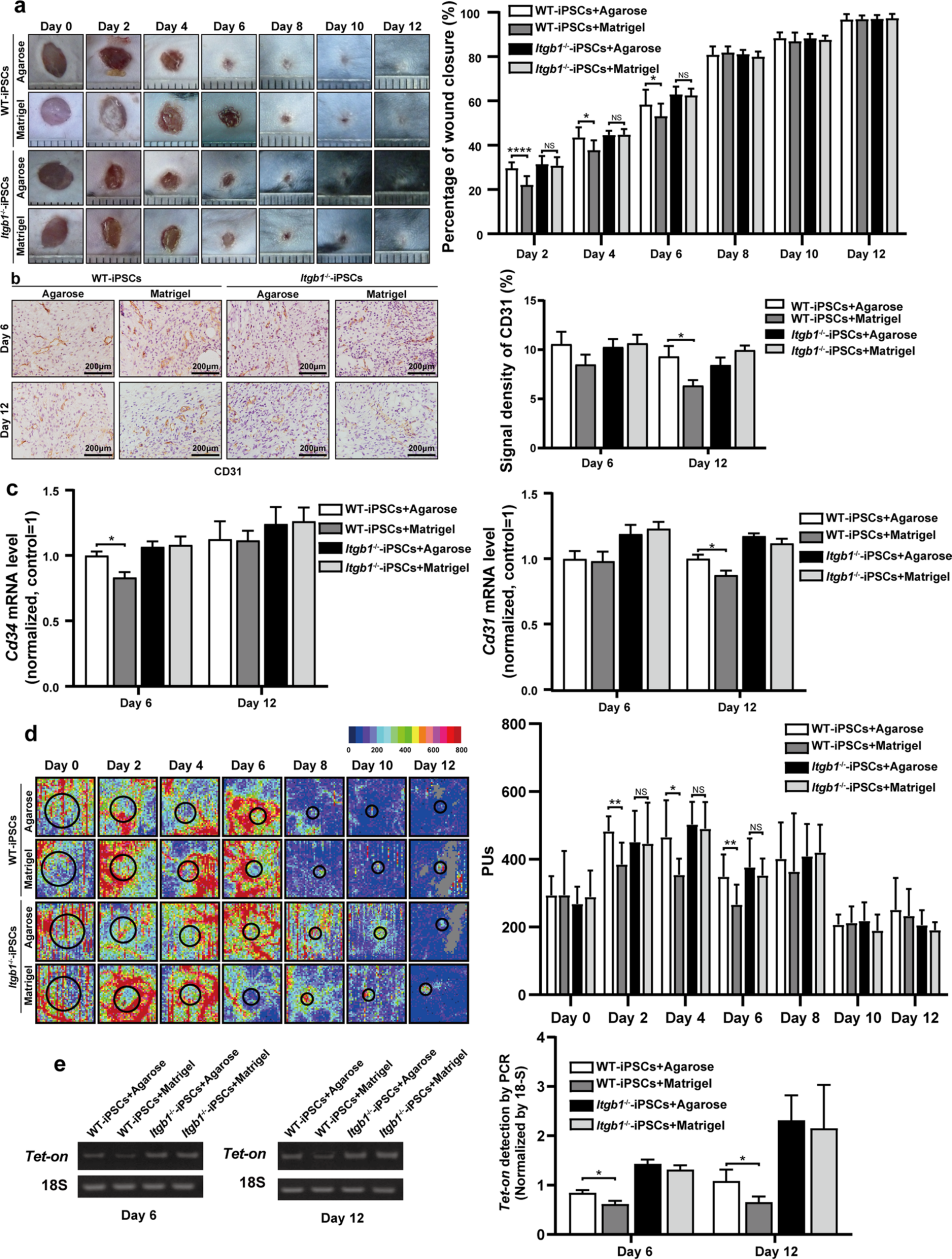
Effects of *Itgb1*-knockout on Matrigel-embedded iPSC-mediated wound healing and iPSC survival in skin wound tissues. **a** Representative macroscopic images of cutaneous wounds treated with Matrigel- or agarose-embedded iPSCs on days 0, 2, 4, 6, 8, 10, and 12 post-treatment (left). Quantification of wound healing expressed as the percentage of healing relative to initial wound size at day 0 (right) (*n* = 10). **b** Representative photomicrographs of CD31 IHC staining of wound tissues on days 6 and 12 post-treatment (left). CD31 signal densities were determined using ImageJ software (right) (*n* = 5). **c** Quantification of *Cd34* and *Cd31* mRNA levels in wound tissues (*n* = 10). **d** Representative blood perfusion images of cutaneous wounds treated with iPSCs or an equal volume of PBS on days 0, 2, 4, 6, 8, 10, and 12 post-treatment (left). Quantification of blood perfusion in wound tissues (right) (*n* = 10). **e** Representative agarose gel electrophoresis images of *Tet-On* and 18S DNA (left). Quantification of *Tet-on* expression normalized against 18S expression in wound tissues (right) (*n* = 10). *p < 0.05, **p < 0.01, ***p < 0.0001

We next used Matrigel to simulate ECM, with agarose as a nonprotein matrix control, to quantify the effect of the ECM on healing. We created a full-thickness excisional cutaneous wound on the dorsal skin of mice and transplanted iPSCs embedded in Matrigel or agarose topically, followed by macroscopic evaluation 2 days later to quantify wound healing. Healing with Matrigel-embedded WT-iPSCs was significantly decreased relative to that with agarose-embedded WT-iPSCs on days 2 (22.14 ± 3.69% vs. 29.59 ± 2.54%) and 4 (37.78 ± 4.18% vs. 43.51 ± 4.41%) post-treatment, whereas we observed no significant difference between Matrigel- and agarose-embedded *Itgb1*^−/−^-iPSC treatment groups on any day (Fig. 5a).

### Absence of ITGß1 rescues Matrigel-mediated inhibition of accelerated angiogenesis and blood perfusion

We next performed IHC staining for CD31 to assess the effect of the ECM on angiogenesis at days 6 and 12 post-treatment. CD31 expression with Matrigel-embedded WT-iPSC treatment was significantly lower than that with agarose-embedded WT-iPSC treatment on day 12 (6.36 ± 1.09% vs. 9.30 ± 2.14%), whereas there was no significant difference in CD31 expression between Matrigel-embedded *Itgb1*^−/−^-iPSC and agarose-embedded WT-iPSC treatments on days 6 and 12 post-treatment (Fig. 5b). Quantitative RT-PCR revealed significantly lower *Cd34* expression with Matrigel-embedded WT-iPSC treatment relative to that with agarose-embedded WT-iPSC treatment on day 6 (0.83 ± 0.12 vs. 1.00 ± 0.09) post-treatment, whereas there was no significant difference observed between the Matrigel-embedded *Itgb1*^−/−^-iPSCs and agarose-embedded WT-iPSC treatments on either day (Fig. 5c, left panel). Moreover, relative *Cd31* expression with Matrigel-embedded WT-iPSC treatment was significantly lower than that with agarose-embedded WT-iPSC treatment on day 12 (0.88 ± 0.10 vs. 1.00 ± 0.09) post-treatment, whereas no significant difference was observed between the Matrigel-embedded and agarose-embedded *Itgb1*^−/−^-iPSC treatments on days 6 and 12 post-treatment (Fig. 5c, right panel).

We then performed serial laser Doppler imaging of the dorsal skin of mice to measure iPSC-mediated acceleration of blood perfusion during wound healing. Perfusion with Matrigel-embedded WT-iPSC treatment was significantly lower than that with agarose-embedded WT-iPSC treatment on days 2 (385.20 ± 60.75 PUs vs. 483.20 ± 41.80 PUs) and 4 (354.10 ± 45.25 PUs vs. 465.40 ± 103.32 PUs) post-treatment, whereas no significant difference in perfusion was observed between the Matrigel-embedded and agarose-embedded *Itgb1*^−/−^-iPSC treatments on any day post-treatment (Fig. 5d).

### Absence of ITGß1 rescues Matrigel-mediated inhibition of iPSC survival in skin wounds

We then measured *Tet-on* expression to evaluate iPSC survival on days 6 and 12 post-treatment. We found significantly lower expression with Matrigel-embedded WT-iPSC treatment was significantly lower than that with agarose-embedded WT-iPSC treatment on days 6 (0.62 ± 0.19 vs. 0.84 ± 0.17) and 12 (0.66 ± 0.34 vs. 1.08 ± 0.68), whereas no significant difference was observed between the Matrigel-embedded and agarose-embedded *Itgb1*^−/−^-iPSC treatments on either day (Fig. 5e).

### Tracking of fluorescently labeled cells in skin wounds

We used iPSCs labeled using PKH26- and GFP-overexpressing iPSCs to detect their presence in the skin wounds at days 6 and 12. Although fluorescent cells were identified, they were difficult to quantify because of their uneven distribution; moreover, they were difficult to differentiate from autofluorescence, regardless of *Itgb1* genotype, Matrigel embedding, or agarose embedding (Additional file 1: Figs. S1 and S2).

### Absence of the integrin alpha-4 subunit alters neither iPSC behavior nor therapeutic efficacy

To test for a role for integrin alpha subunits, we knocked out *Itga4* in iPSCs using CRISPR-Cas9; the targeting was confirmed by DNA sequencing (Additional file 1: Fig. S3). The absence of ITGα4 had no effect on iPSC pluripotency, function, or survival in culture (Additional file 1: Figs. S3, S4 and S5); nor did it affect wound healing, angiogenesis, blood perfusion, or survival in skin wounds in vivo (Additional file 1: Figs. S1, S2 and S6), independent of Matrigel or agarose-embedding.

## Discussion

The therapeutic potential of iPSCs is enormous [17]. We have previously demonstrated that topical administration of iPSCs to treat skin wounds promotes angiogenesis, accelerates wound healing, and avoids the side effect of teratoma formation [18]. However, the extent of the therapeutic efficacy of these administered cells remains limited to the site on which the cells are delivered, which also limits the distance through which they can act [19, 20]. Previous reports indicate that integrins mediate adhesion between stem cells and ECM [21–23]; we previously reported that ITGβ1 is expressed at high levels on the surface of iPSCs. The expression level of β1 was more than 1000-fold higher than that of β2 [8]. We hypothesized that although stem cell–ECM adhesion mediates stem-cell anchoring, adhesion also decreases the migratory potential of stem cells, thereby reducing their area of action. Furthermore, ITGβ1 inhibition or gene knockout reduces iPSC adhesion to the ECM, extends iPSCs distribution in skin tissues, and promotes wound healing.

The mechanisms by which iPSCs function in cutaneous wound healing include accelerating re-epithelialization and promoting angiogenesis and blood perfusion [8]. In the present study, the *Itgb1* knockout had no influence on pluripotency or in vitro function in the absence of Matrigel. Using a full-thickness excisional cutaneous wound model, we found that topical administration of iPSCs promoted early-stage (days 2–6 post-treatment) wound healing, angiogenesis, blood perfusion, and iPSCs survival with WT-iPSC treatment relative to controls and that these activities were significantly enhanced by *Itgb1*^−/−^-iPSCs relative to the WT-iPSCs. During wound healing, blood flow changes over time. High perfusion occurs in the early stage of wound repair. With the granulation tissue remodeled into a scar in the advanced stage of wound repair, blood flow decreases to normal levels gradually, because in normal tissue only a portion of capillaries open under the control of capillary sphincters. Thus, at the time that the treated lesions had finished repairing while untreated lesions had not, blood perfusion in untreated lesions was expected to be higher.

The clinical efficacy of stem-cell transplantation depends on the quantity of stem cells needed to treat damaged sites; therefore, the development of more efficient methods for their delivery is essential for enhancing the survival of transplanted cells and maximizing their therapeutic efficacy [24, 25]. A previous study reported that ITGβ1 plays an essential role in adhesion to the ECM and adjacent cells, serving as the link between extracellular molecules and the intracellular cytoskeleton [26]. In the present study, we showed that knocking out *Itgb1* improved the efficacy of iPSCs in accelerating cutaneous wound healing, suggesting that ITGβ1 mediates interactions between transplanted iPSCs and the ECM, limiting the extent of iPSC activity to the site of their delivery.

To investigate the effect of ITGβ1-mediated cell-ECM interaction on cutaneous wound healing, we used Matrigel to simulate the ECM, with agarose as a nonprotein matrix control, and compared their effects on WT and *Itgb1-*knockout iPSCs. Agarose gel has a similar pore size and stiffness as Matrigel, but does not contain ITGβ1-binding sites. We observed significantly lower levels of wound healing, angiogenesis, blood perfusion, and iPSC survival with Matrigel embedding than with agarose embedding during early wound healing (days 2–6) whereas we observed no significant differences between Matrigel and agarose embedding of *Itgb1*^−/−^-iPSCs throughout healing. Our results suggest that the absence of ITGβ1 is beneficial in wound healing when cells are applied topically. When embedded in agarose gel, the effect of iPSCs was not altered by knocking out *Itgb1*. Since collagen is a primary ligand of integrin β1 in the ECM, we used it as a known ligand control, in addition to agarose. The effects of collagen and Matrigel embedding were similar, further confirming the importance of the presence of ITGβ1-binding sites.

Our result showed knockout of integrin α4 did not change the interaction between iPSCs and ECM; nor did it influence the therapeutic efficacy in our model.

The high apoptosis rate of transplanted stem cells in the niche of recipient tissue is a major problem. Integrin-mediated adhesion between cells and the ECM promotes cell migration [27, 28]. Our in vitro data showed that Matrigel promotes migration and increases apoptosis in *Itgb1*^−/−^-iPSCs compared to WT-iPSCs, which may lead to a decreased presence of *Itgb1*^−/−^-iPSCs in wounds. However, our in vivo experiments showed that *Itgb1*^−/−^-iPSCs are more abundant than WT-iPSCs in lesions. Cell survival depends on both cell-intrinsic factors and microenvironment. Because the *Itgb1* knockout promotes cell migration, the topically applied iPSCs leave the wound surface earlier and migrated more deeply. Therefore, such iPSCs may inhabit a microenvironment that better promotes their survival.

We tried to track the fate of fluorescently labeled iPSCs in the wound lesions, but were unsuccessful. It is possible that the fluorescence faded and/or cells expressing GFP were killed by antibodies against GFP. The possibility of being killed by antibodies against GFP is supported by the existence of many commercially available mouse antibodies against GFP. It was also one of the reasons that we did not use GFP-expressing iPSCs for the main experiment.

Stem cells have been widely used in wound healing. The stromal vascular fraction (SVF) containing adipose stem cells (ASCs) has been routinely used for regenerative medicine and surgical applications. ASC-based therapies could be based on the use of decellularized ECM as a scaffold re-cellularized by ASCs of the recipient, as the safety and efficacy of allogenic ASCs and ECM transplants have been demonstrated without major side effects [29, 30]. Tissue bioengineering is an ideal therapeutic approach to wound healing and soft-tissue defects; platelet-rich plasma (PRP) plays an important role, as the PRP/media ratio provides sufficient nutrition for transported cells. A bio-functionalized scaffold composed of PRP and hyaluronic acid (HA) treatment showed better regenerative potential than did HA alone in terms of re-epithelialization and dermal regeneration [31–33]. The efficacy of PRP + HA has been demonstrated without complications [34]. Mesenchymal stem cells (MSCs) enhance angiogenesis in vivo on 3D ECM-based microgel platform [35]. ECM proteins directly interact with cell surface integrins to modulate of the performance of mesenchymal stem cell and promote wound re-epithelialization [36, 37]. Adipose-derived mesenchymal stem cells (AD-MSCs) are easy to obtain from various fat depots and show potential to generate cells involved in wound healing. It has been demonstrated that the application of AD-MSCs, PRP, and biomaterials to soft-tissue defects is safe and efficient and without major side effects [38].

Hair regrowth plays an important role in hard-tissue wounds, necessitating hair bioengineering. Human hair-follicle mesenchymal stem cells (HF-MSCs) contained in micrografts increase both hair count and density and may represent a safe and viable treatment for hair loss [39]. Hair follicle stem cells (HFSCs) promote hair regrowth and improve hair density. HFSC scalp treatment is safe and feasible in clinical preliminary studies [40, 41]. PRP and HF-MSC-derived signaling and growth factors influence hair growth through cellular proliferation to prolong the anagen phase (FGF-7), induce cell growth (ERK activation), stimulate hair-follicle development (β-catenin), and suppress apoptotic cues (Bcl-2 release and Akt activation) [42, 43]. PRP alleviated androgenetic alopecia without major side effects, so can be considered as a safe and effective treatment for hair loss [44]. PRP, HFSCs, and adipose-derived stem cells (ASCs) are considered to be effective in autologous stem cell-based therapy for hair regrowth in patients affected by androgenetic alopecia, as well as for wound healing [45, 46].

Stem cells also play an important role in soft-tissue defects. Human ASCs systematically characterized for growth features, phenotype and multipotency in soft tissue defects, can provide new therapeutic approaches to breast reconstruction [47]. Adipose-derived stromal vascular fraction (AD-SVFs) and adult adipose-derived mesenchymal stem cells (AD-MSCs) can be used for autologous therapies [48]. MSCs could represent an effective, autologous and safe therapy for coronavirus infection (COVID-19). ASCs have been identified as a new regenerative immediate therapy for COVID-19-induced pneumonia [49]. AD-MSCs have the potential to reduce the risk of complications and death due to their strong immunomodulatory nature, which can improve microenvironment, promote neovascularization, and enhance tissue repair capabilities in COVID-19-induced pneumonia [50–52].

Our strategy for promoting therapeutic efficacy by decreasing adhesion between stem cells and ECM, therefore increasing stem-cell migration through the ECM, may also apply to stem cell types other than iPSCs and tissue injuries other than skin wounds.

## Conclusions

We have shown that *Itgb1* knockout in a mouse cutaneous skin-wound model promoted the therapeutic effects of iPSCs, including improved skin-wound healing via increased angiogenesis, blood perfusion, and iPSCs survival. Our data suggest that the *Itgb1* knockout freed iPSCs from the restriction of their adhesion to the ECM, which increased their migration and facilitated the transfer of additional iPSCs to the target site. These findings offer a new strategy for promoting the effectiveness of iPSCs in skin-wound treatment.

## Supplementary Information

**Additional file 1:** Supplemental material.

## Data Availability

The datasets used and analyzed during this study are available from the corresponding author on reasonable request.

## Abbreviations

AP: Alkaline phosphatase
CCK-8: Cell Counting Kit-8
DMEM: Dulbecco’s modified Eagle’s medium
EB: Embryoid body
ECM: Extracellular matrix
FBS: Fetal bovine serum
iPSCs: Induced pluripotent stem cells
ITGβ1/*Itgb1*: Integrin β1
ITGα4/*Itga4*: Integrin α4
KLF4: Krüppel-like factor 4
LIF: Leukemia inhibitory factor
NEAA: Nonessential amino acid
Oct4: Octamer-binding transcription factor 4
PBS: Phosphate-buffered saline
RT: Reverse transcription
Sox2: Sex-determining region Y-box 2
Ssea1: Stage-specific embryonic antigen 1
