# Supplementary material for: Knockout of integrin β1 in induced pluripotent stem cells accelerates skin-wound healing by promoting cell migration in extracellular matrix
Source: Stem Cell Res Ther. 2022 Jul 30;13:389. doi: 10.1186/s13287-022-03085-7 (PMC9338467; doi:10.1186/s13287-022-03085-7)
Supplement: Supplementary file 1 — Additional file 1: Supplemental material. [file 13287_2022_3085_MOESM1_ESM.pdf]

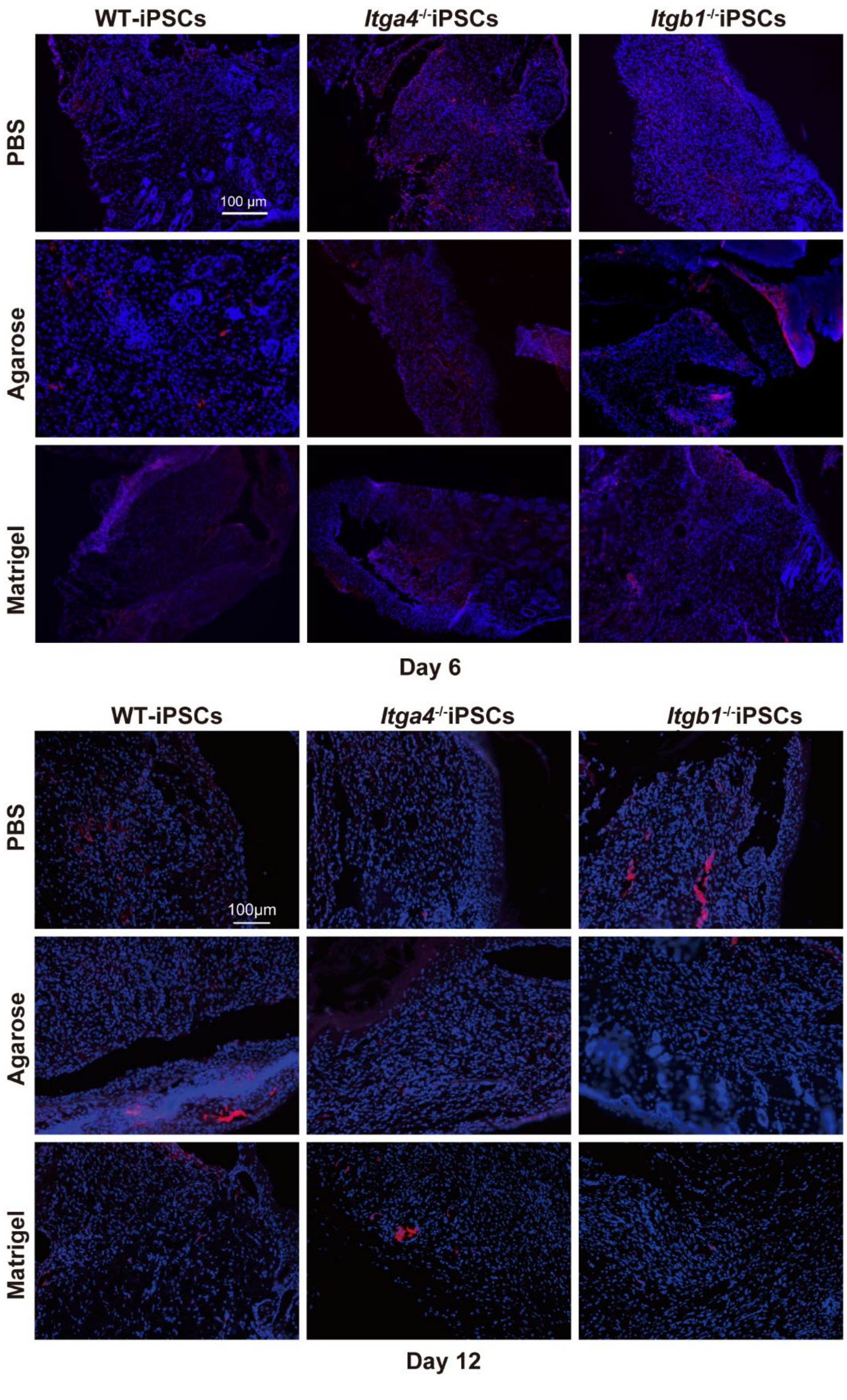

Figure S1. PKH26 fluorescence in wound sections on days 6 and 12.

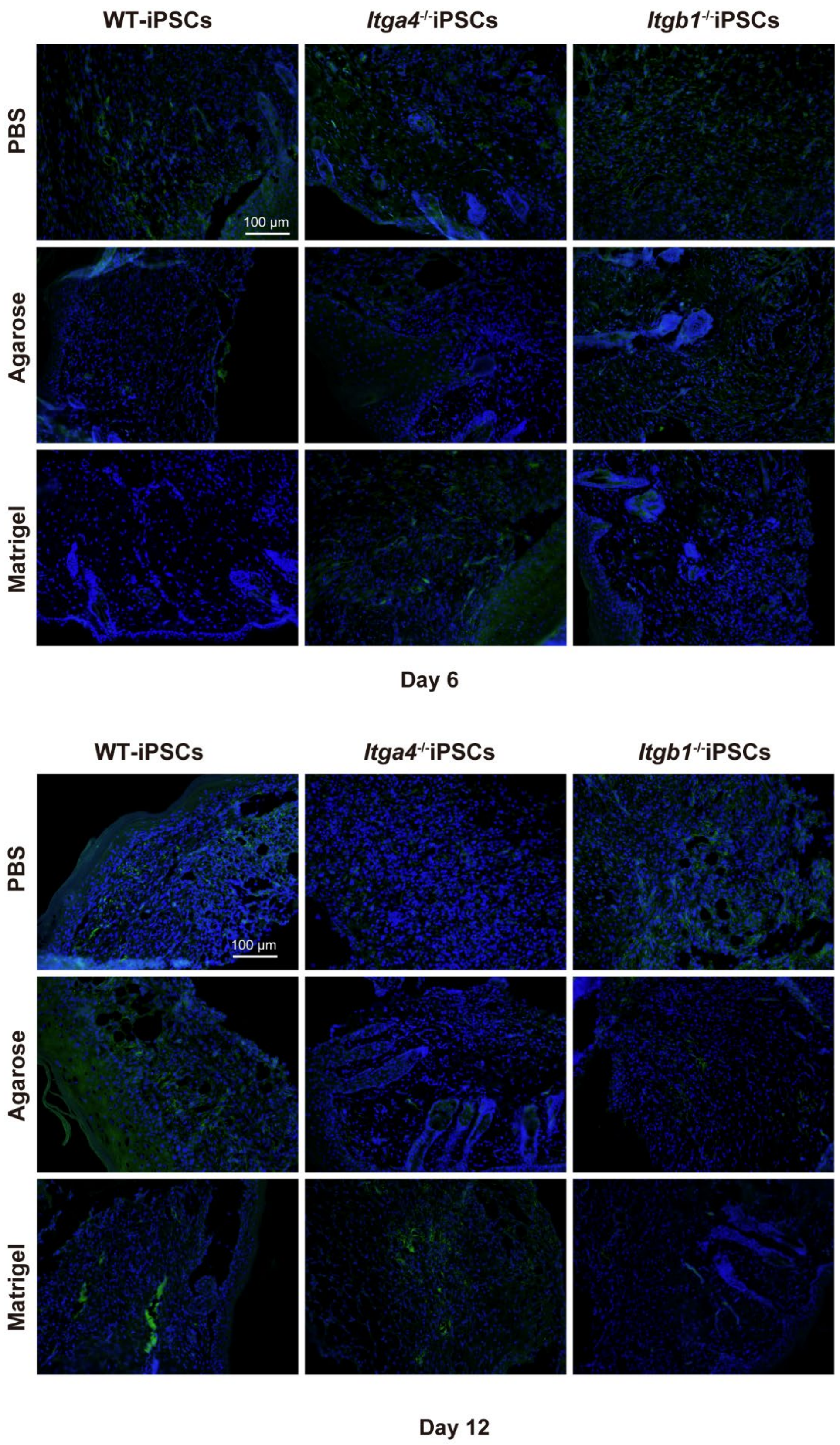

Figure S2. GFP expression in wound sections on days 6 and 12.

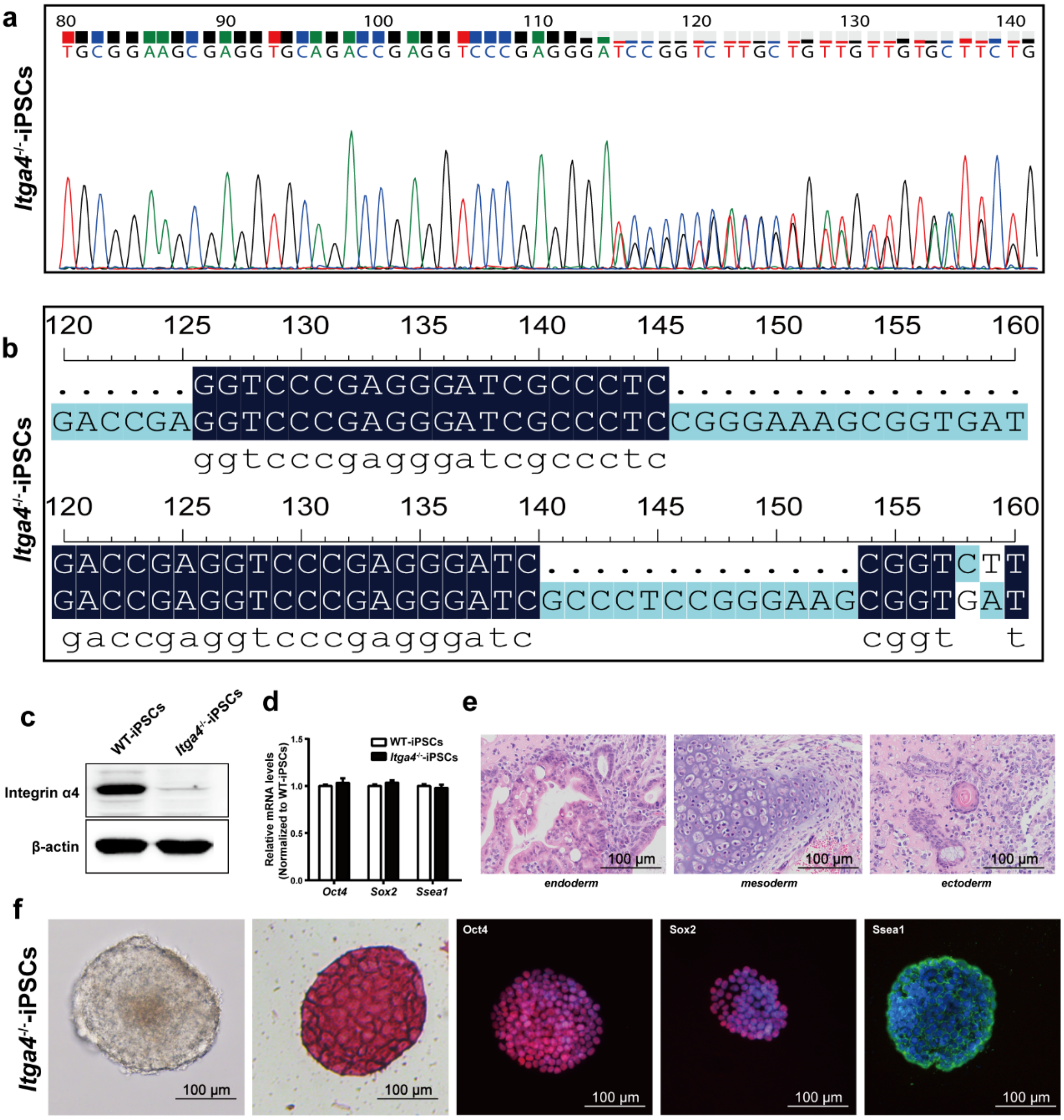

Figure S3. Production and characterization of *Itga4*-knockout iPSCs. **a-b** DNA sequencing and pairwise dot matrix comparison of *Itga4*$^{-/-}$-iPSCs showing mismatch loss. **c** Western blot confirming *Itga4* knockout in iPSCs. **d–f** *Itga4*$^{-/-}$-iPSCs formed EBs and teratomas, exhibited AP activity, and expressed pluripotency mRNA and protein markers.

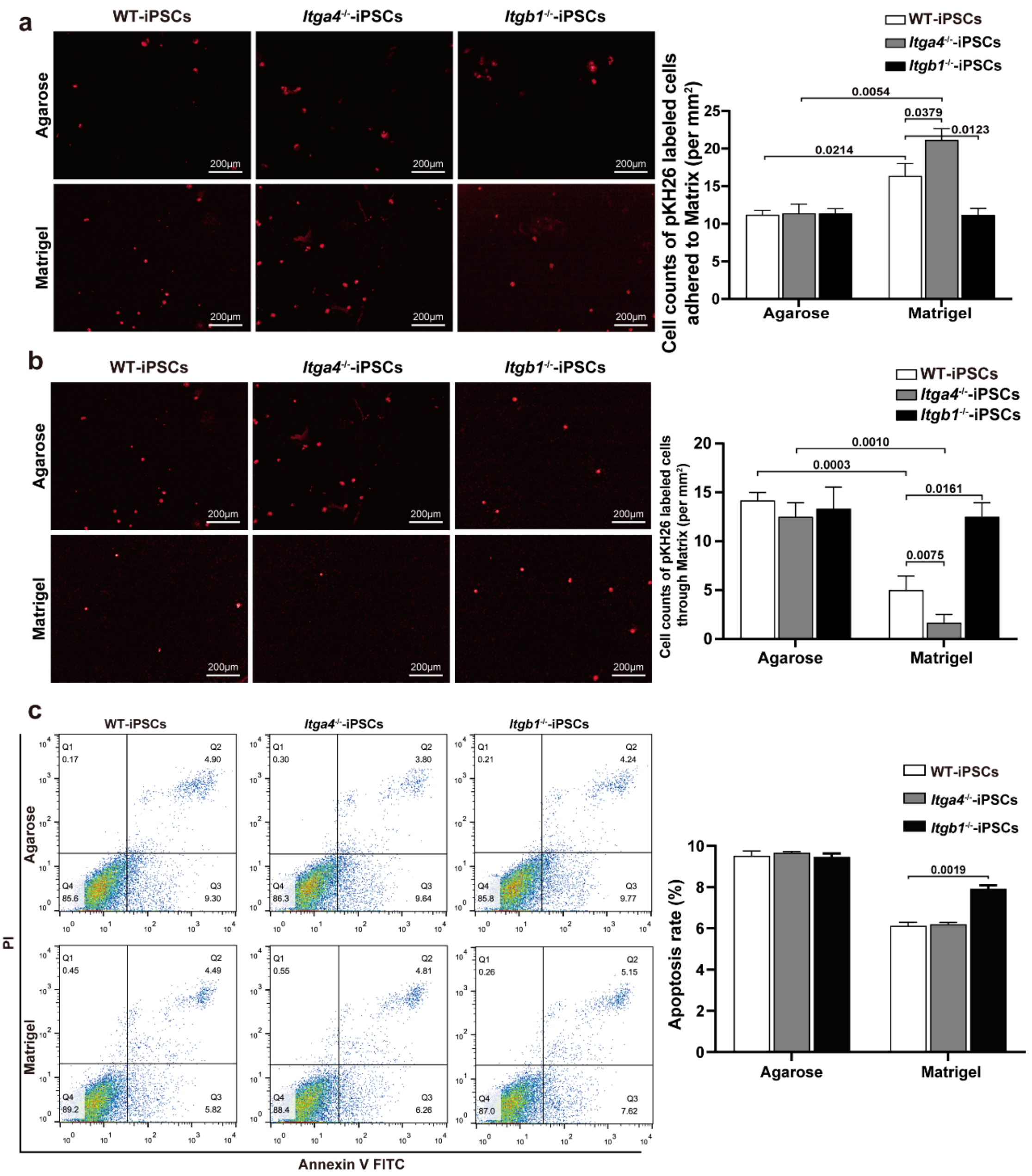

Figure S4. Effects of *Itga4* knockout on the function and survival of iPSCs cultured on Matrigel. **a** Representative fluorescence imaging (left) and quantification (right) of PKH26-labeled iPSCs adhered to plates with agarose (top row) and Matrigel (bottom row) (n = 5). **b** Representative fluorescence imaging (left) and quantification (right) of PKH26-labeled iPSC migration across the chamber membrane in the presence of agarose, collagen and Matrigel (n = 3). **c** Apoptosis measured by Annexin V/FITC staining (left) and quantification (right) of iPSC in the presence of agarose, collagen and Matrigel (n = 3).

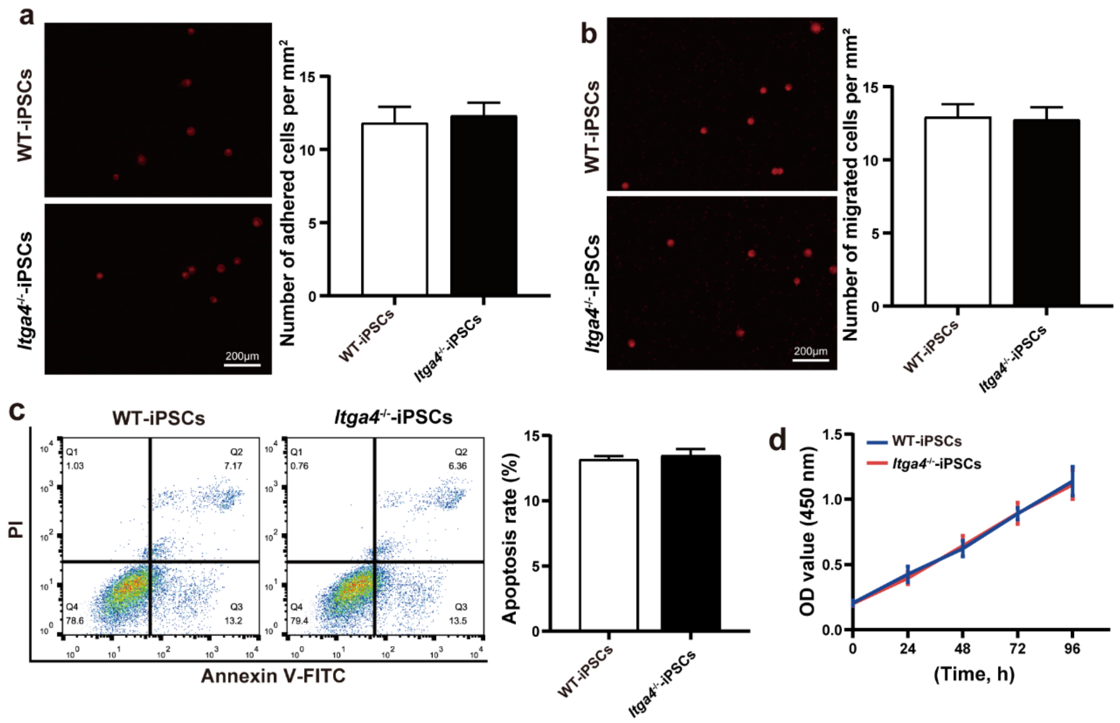

Figure S5 Effects of *Itga4* knockout on iPSC function and survival in the absence of Matrigel. **a** Representative fluorescence imaging (left) and quantification (right) of PKH26-labeled iPSCs grown in plates without Matrigel (n = 5). **b** Representative fluorescence imaging (left) and quantification (right) of PKH26-labeled iPSC migration across the chamber membrane in the absence of Matrigel (n = 3). **c** Levels of iPSC apoptosis in the absence of Matrigel determined by Annexin V/FITC staining (n = 3). **d** iPSC proliferations in the absence of Matrigel determined by CCK-8 assay (n = 6).

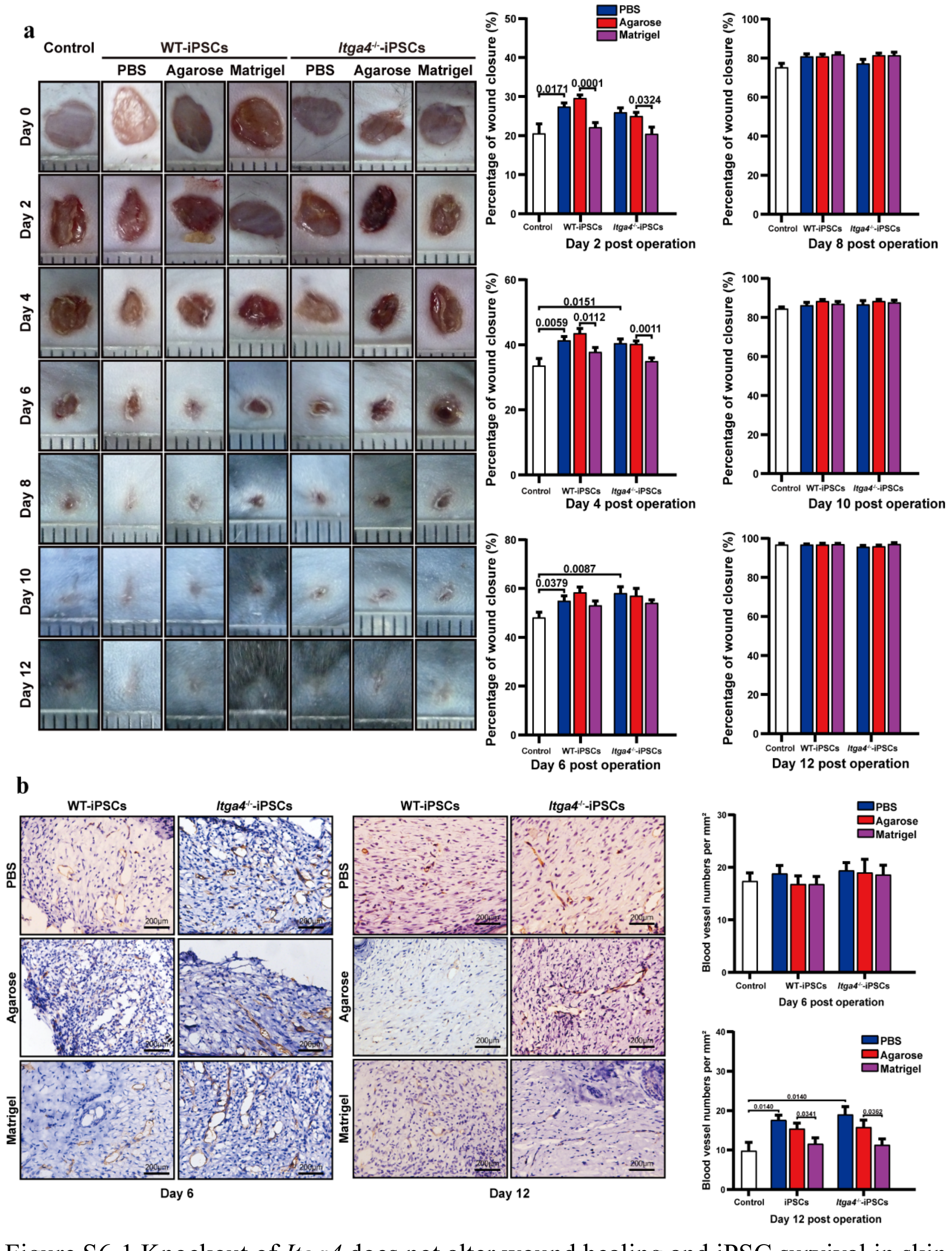

Figure S6-1 Knockout of *Itga4* does not alter wound healing and iPSC survival in skin wounds. **a** Representative macroscopic images of cutaneous wounds treated with (agarose-embedded, Matrigel-embedded or PBS diluted) WT-iPSCs and *Itga4*$^{-/-}$-iPSCs on days 0, 2, 4, 6, 8, 10, and 12 post-treatment (left). Quantification of wound healing expressed as the percentage of healing at each time point relative to the wound size at day 0 (right) (n = 10). **b** Representative photomicrographs of CD31 IHC staining of wound tissues on days 6 and 12 post-treatment (left). CD31 signal densities quantified using ImageJ software (right) (n = 10).

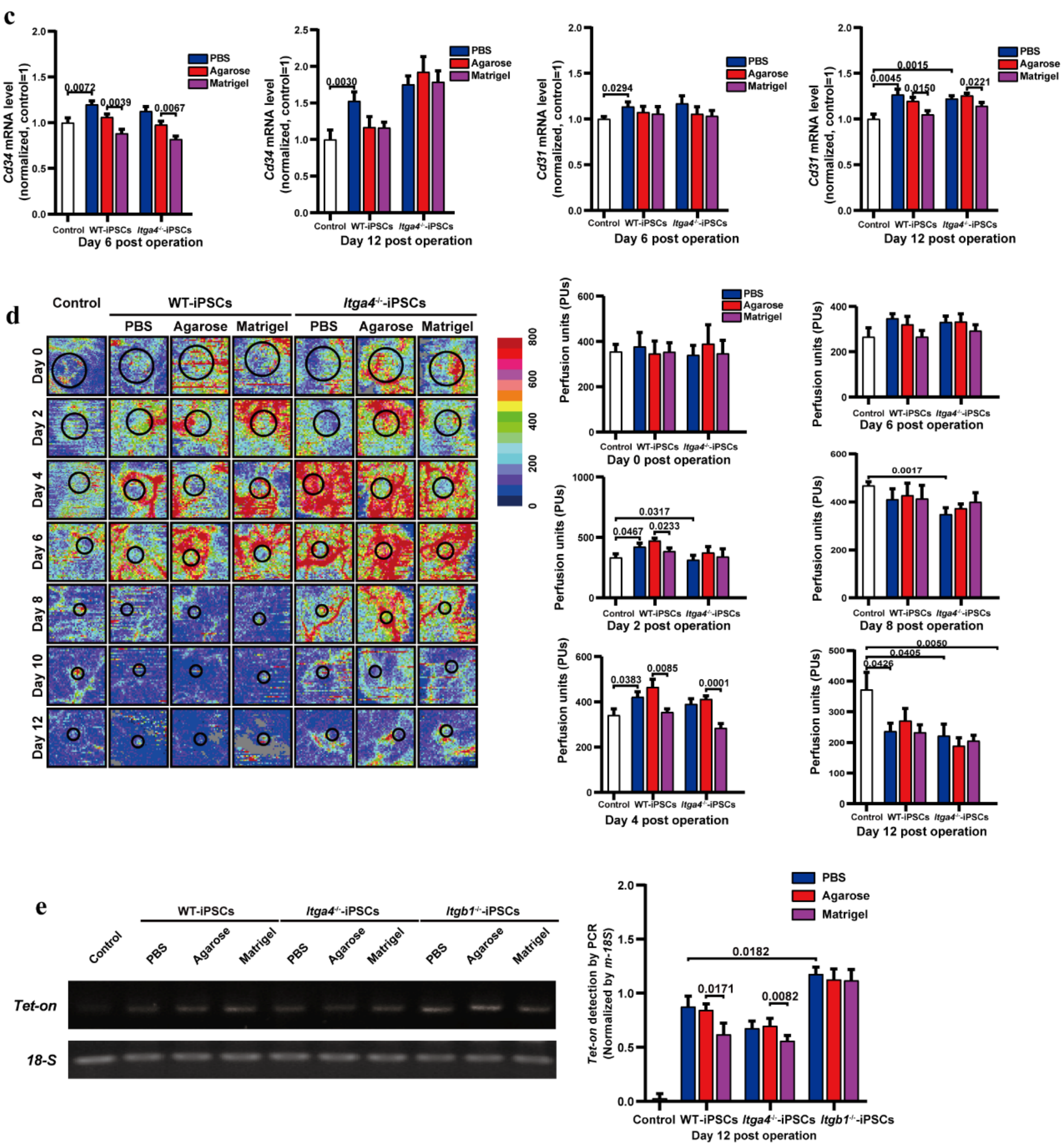

Figure S6-2 Knockout of *Itga4* does not influence wound healing and iPSC survival in skin wounds. **c** Quantification of *Cd34* and *Cd31* mRNA levels in wound tissues (n = 10). **d** Representative blood perfusion images of cutaneous wounds treated with iPSCs or an equal volume of PBS on days 0, 2, 4, 6, 8, 10, and 12 post-treatment (left). Quantification of blood perfusion in wound tissues (right) (n = 10). **e** Representative agarose gel electrophoresis images of *Tet-On* and 18S DNA (left). Quantification of *Tet-on* expression normalized against 18S expression in wound tissues (right) (n = 10).
